# The Annual Commencement of the Medical Department of Niagara University

**Published:** 1887-04

**Authors:** 


### *THE ANNUAL COMMENCEMENT OF THE MEDICAL DEPARTMENT OF NIAGARA UNIVERSITY.*

The annual commencement of the college will take place April 12, 1887. The annual meeting of the alumni will also be held on the same day. No pains have been spared to make the occasion a pleasant and profitable one to those who attend. The school, as has been announced, is in a most flourishing con dition, there being a good class in attendance. Considering the high standard of requirements, it is certainly marvelous that this institution has succeeded so well. It is learned that about one-tenth of the class will be candidates for graduation; that two of those who have presented themselves for graduation have attended four full courses of lectures; that several now in the school will take four years by choice. Too much cannot be said in praise of such a course on the part of the teachers and students of this institution. The profession generally sustain this college, and will continue so to do.

The exercises, a programme of which has been sent us, will take place April 12, beginning at 11 A.M. Morning session of the Alumni Association will be held at this hour at the college building, on Ellicott street, near Broadway, at which time Dr. William H. Heath, president of the association, will deliver an address, followed by the regular order of business and the election of officers for the ensuing year. The afternoon session will be held at the college building at 3 P. M. Dr. Simeon T. Clark, of Lockport, professor of medical jurisprudence in the college, will give the address ot welcome. Dr. Floyd S. Crego will read a paper on "Defective Mental Inhibition." Dr. Stephen Smith, of New York City, the celebrated author of the work on "Operative Surgery," and an eminent teacher, will read a paper on "The Influence of Antisepsis in Surgery on Human Longevity." Dr. Henry D. Ingraham will read a paper on "Intubation of the Larynx," and Dr. Frank H. Potter "Some General Rules Governing Operations in the Nasal Passages." In the evening, the commencement exercises will be held. The university rules follow the English method of conferring degrees. The candidates will be hooded and degrees conferred by the Chancellor. Rev. E. Dodge, D. D., LL. D., president of Madison University, will deliver the address of the evening. Prof. A. R. Davidson, of the faculty, will address the graduates. Following this, the annual banquet will be held, at which several toasts will be responded to, and music will be furnished by the College Glee Club.

The executive committee will issue invitations to the profession to be present at any or all of these meetings. They desire us also to extend, through these columns, a general invitation to all to be present, which we most heartily do. It is hoped by the committee that many will avail themselves of the opportunity to meet Dr. Stephen Smith and attend the exercises in the evening. Physicians from out of town will be especially welcome. A large number have already signified their intention to be present. Comfortable apartments will be secured for those desiring them if they will send word a few days in advance to Dr. A. A. Hubbell, Buffalo, N. Y.

---

Dr. Edward Clark, demonstrator of anatomy in the Medical Department of Niagara University, leaves the city to spend a few months in New York City, for the special purpose of studying diseases of the rectum, under the instruction of Dr. Chas. B. Kelsey, who has acquired a large reputation in this specialty. We tender to our wide-awake and energetic friend our warmest approval for the effort he is putting forth in an important field of professional study. Few bring to this work such an aptitude and preparation upon which to build up a thorough scientific knowledge of a special class of diseases as Dr. Clark. He has filled a difficult position in the college with marked ability. As demonstrator and lecturer on anatomy, he has given the best guaranty of fitness by achieving a measure of success which few young men are permitted to secure, either by their natural ability or through their professional acquirements. We believe Dr. Clark has a bright future before him. His present purpose shows that he has the willingness to grasp at opportunities from which well-earned success will surely follow.

---

We call attention to the advertisement of the Buffalo Maternity Hospital, among our advertising pages. This institution, organized in May, 1886, is under the professional charge of our editorial confrere, Dr. Lothrop, professor of obstetrics in the Medical Department of Niagara University, and his able assistant, Dr. C. C. Fredericks, associate editor of this Journal. An able corps of medical men form the consulting staff. The names of the attending and consulting staff are a guaranty of the high character of the institution. The success of the hospital has exceeded the expectations of its projectors, and it has already become one of the best in this State. That it supplies a want long felt, is assured by the measure of support it has received since its organization. We bespeak for it the endorsement of the profession, to whom we commend the hospital for its earnest efforts in behalf of a class of patients who require exceptional skill in their management, and the kindest and most considerate care for a successful puerperal convalescence.

---

We congratulate the faculty of the *Buffalo Medical College* upon the large class graduated and on the fact that not a single graduation fee had to be returned. Of the entire class of fifty, not one failed to pass the examination for the doctorate. The alumni will rejoice at this evidence of the superior mental ability of the young men in attendance, in spite of the fact that all who present themselves are received as students without any inquiry as to preliminary education. We can hardly credit the report that the faculty were so gratified with the attainment of the members of the class, that it was proposed in addition to the M. D. to confer upon each of them the degree of M. A.; although a precedent for such action was established by conferring the degree of B. S. upon one of the teachers of the school. It is stated that the college, by the provisions of its charter, can grant any degree. If in the future the laws of the State should demand that candidates for the medical degree should possess a degree in arts, provision will doubtless be made to comply with the law.

---

We are glad to welcome the publication of the *Journal of Laryngology and Rhinology*. It is edited by Morell MacKenzie, M. D., and R. Norris Wolfenden, M. D., of London, and will furnish each month an analytical record of the current literature relating to the throat and nose. This will be invaluable for those engaged in the practice of the specialty. There are associated with the editors prominent specialists from each of the principal countries of Europe, together with one each from Australia and America, who will furnish the current news for their respective countries. This country has a most fitting representative in Dr. John N. Ma‿Kenzie, of Baltimore. We bespeak for the *Journal* a large circulation.

---

THE American System of Gynecology, which for some time past has figured among the more important announcements of Messrs. Lea Brothers & Co., of Philadelphia, we are glad to learn, is well through the press, and may be expected shortly. Numbering among its contributors such prominent authorities as Professors Barker, Battey, Englemann, Garrigues, Goodell, Reeves Jackson, Lusk, Mundé, Reamy, Thomas, Van de Warker, etc., it will certainly present a thoroughly satisfactory and complete statement of the science in its most recent aspects, and we feel justified in congratulating the profession that what has been peculiarly an American specialty is about to receive from American hands the literary tribute due to it.

